# Optimization of Micro-Drilling of Laminated Aluminum Composite Panel (Al–PE) Using Taguchi Orthogonal Array Design

**DOI:** 10.3390/ma16134528

**Published:** 2023-06-22

**Authors:** Bekir Yalçın, Ali Yüksel, Kubilay Aslantaş, Oguzhan Der, Ali Ercetin

**Affiliations:** 1 Department of Mechanical Engineering, Faculty of Technology, Afyon Kocatepe University, Afyonkarahisar 03200, Turkey; aliiyuksel@yandex.com (A.Y.); aslantas@aku.edu.tr (K.A.); 2 Department of Marine Vehicles Management Engineering, Maritime Faculty, Bandırma Onyedi Eylul University, Bandırma 10200, Turkey; oder@bandirma.edu.tr; 3 Department of Naval Architecture and Marine Engineering, Maritime Faculty, Bandırma Onyedi Eylul University, Bandırma 10200, Turkey; aercetin@bandirma.edu.tr

**Keywords:** aluminum, composite panel, micro-drilling, cutting force

## Abstract

Aluminum Matrix Composite (AMC) represents an innovative class of materials that is extensively utilized in industries such as automotive, defense, aerospace, structural engineering, sports, and electronics. This study investigates the thrust force, exit burr formation, changes in the micro-tool, and drilled hole diameters during the micro-drilling of an aluminum-polyethylene composite panel (Al–PE). The panel consists of 3501 series aluminum skin materials bonded to a polyethylene (PE) core. Micro-drilling test parameters were designed using Taguchi’s L16 (4^2^ 2^3^) orthogonal array. Tests were conducted with five control parameters: cutting speed with four levels (10 m/min, 20 m/min, 30 m/min, 40 m/min), feed rate with four levels (0.5 µm/rev, 1 µm/rev, 2 µm/rev, 4 µm/rev), the tool diameter with two levels (0.7 mm, 1 mm), and tool point angle with two levels (100°, 140°) using both AlTiN-coated and uncoated drills. The maximum thrust force (F_z_), maximum burr height, and changes in both the drill tool and hole diameters were measured for analysis of variance (ANOVA). The results showed that, in terms of impact on F_z_, tool point angle had the highest positive influence (64.54%) on the micro-drill at the entrance of composite (upper aluminum plate). The cutting speed had the highest positive influence (45.32%) on the tool in the core layer (PE core layer). The tool point angle also had the highest positive influence (68.95%) on the micro-drill at the lower layer of the composite (the lower aluminum plate). There was noticeable chip adhesion on the major cutting edge and nose area under micro-drilling conditions with higher thrust forces and burr height. The AlTiN coating had a positive effect on tool wear and hole diameter deviations, but it adversely affected the burr height.

## 1. Introduction

Composite materials have been extensively used in many advanced industrial applications such as spacecraft, automobiles, defense, building structures, sports, and electronics due to their lightness [1], corrosion [2], thermal resistance [3], high strength [4,5], and good fatigue life [6]. Composite materials comprise three main divisions in applications: particle-reinforced [7], fiber-reinforced [8], and structural laminates [9]. With this classification, composite materials of various properties have been developed for utilization in different fields. Al–PE falls within the category of structural laminated composites and comprises aluminum skin materials bonded to polyethylene (PE) through a rolling process. Al–PE was initially developed in 1965 by the German brand Aruqbang for decorative purposes. Since then, this material has gained widespread usage in fields such as building and construction, automotive, transportation, railway vehicles, and trailers [10]. This can be attributed to its desirable properties such as durability, strength, adaptability, superior corrosion resistance, lightness, and insulation. This laminated Al–PE composite also undergoes a drilling process for mounting applications. Numerous studies in the literature discuss the mechanical and machining characterization of such materials [11,12,13]. For instance, Gao et al. [14] reported that, when the bonding strength of the steel/Al/steel composite interface is low, delamination first occurs on the exterior of the bending, as seen in their study on the effect of bond strength on the formability of steel/aluminum composite panels. Additionally, Zitoune et al. [15] explained that drilling holes in composite stacks for assembly poses challenges for manufacturing engineers due to the varying machining properties of different materials. Sayer et al. [16] observed that the perforation threshold of hybrid composites impacted by the surface with carbon fiber was approximately 30% higher than that of the surface with glass fiber. In another study [17], it was shown that the thermoelastic performance of aerospace aluminum honeycomb panels allows the panel to cool down uniformly in all three dimensions. Ravishankar asserted that hybrid composites are emerging as an alternative to traditional composites in the global market, especially in automobile applications [18]. Yalçın and Ergene [19] noted that the critical factor for fiber-reinforced laminate composites is the formation of cracks at the interface between the matrix structure and the fibers that carry loads and distribute forces.

Researchers have shown growing interest in micro-manufacturing technology for fabricating miniaturized products made of composite materials in response to escalating industrial demand [20]. Micromachining methods, such as micro-milling [21,22,23], micro-drilling [24], micro-turning [25], micro-grinding [26], laser beam drilling, electrochemical drilling, and ultrasonic drilling [27], are frequently applied to composite materials [28]. The results of both conventional and micro-drilling operations depend on a variety of factors: cutting parameters such as feed rate, cutting speed, and coolant; tool parameters such as helix type, coating type, main angles, and effective edge length [29]; and workpiece material properties, including structure and stacking, strength, hardness, and porosity. However, materials with diverse properties in composite structures can cause cutting difficulties and poor manufacturing quality [30]. Consequently, researchers are striving to make significant progress in the micro-manufacturing of various metal-, ceramic-, polymer-, and composite-based components. Durao et al. [31] stated that composites are non-homogeneous, making drilling difficult due to issues such as delamination, pull-out, inter-laminar cracking, or thermal damage. Their study found that a low feed rate and a suitable tool point angle can help reduce thrust force and delamination. Another study highlighted that delamination problems, such as peeling of the upper layer and pushing out of the uncut layer, can occur due to thermal and cutting forces during the drilling of laminated composite. This phenomenon can lead to deterioration of long-term performance under fatigue loads [32]. Prasanna et al. [33] examined the effect of small-hole drilling on the quality characteristics of a carbon fiber-reinforced polymer composite (CFRP). It was found that the circularity error decreases with a low feed rate, and it was concluded that a higher spindle speed reduces the circularity error and taper. Furthermore, Khan studied the hole quality in CFRP drilling considering multiple performance characteristics, reporting that low torque results in defect-free and high-quality holes [34]. Kim et al. [35] reported that exit burrs of various sizes and shapes form when the drill approaches the bottom of the hole, and stress increases either along the edge or at the center of the hole exit due to tool advancement. Moreover, mechanical deburring of a micro-hole can be very challenging because it is hard to locate the hole, and a chemical deburring process is not suitable as it can distort the hole shape. Therefore, preventing exit burr formation is preferable in micro-drilling [35].

## 2. Thrust Force and Burr Formation in Micro-Drilling

Micro-drilling is a common machining process that takes place in just twenty percent of the time required for traditional processes. It is estimated that around 250 billion drill tools are used annually in the US alone [36]. The need for micro-drilling was first recognized in the 1940s, leading to attempts to develop high-quality micro-tools [37]. The Swiss micro-tool manufacturer, Siphinx, defines micro-drilling as a process that creates holes ranging from 0.05 mm to 2.5 mm in diameter, although no standard definition exists [38]. Micro-drilling shares many similarities with conventional drilling, but the reduction in drill dimensions introduces several problems, including exit burr formation, tool breakage, hole shape distortion, excessive vibration, and tool plowing [39,40]. To understand how the mechanics of micro-drilling differ from conventional drilling, various models of forces in micro-drilling have been developed. For example, Sambhav et al. [41] created an analytical model of the shearing forces and plowing forces exerted by the major cutting edges. Zhang et al. [39] provided a comprehensive summary of the forces at all the cutting elements on each edge and all the cutting edges on the drill.

**Figure 1:**
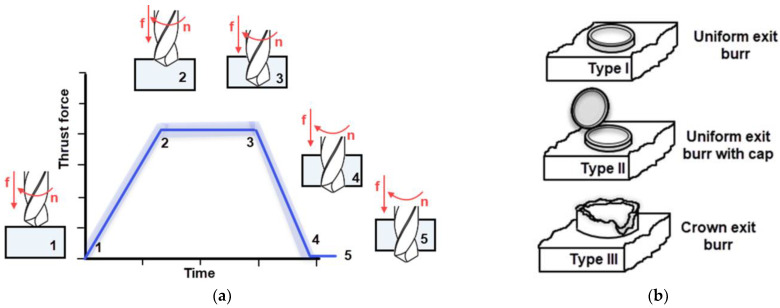
(**a**) A typical profile of the thrust force on major cutting edges in micro-drilling, (**b**) comparative analysis of uniform exit burr, uniform exit burr with cap, and crown exit burr.

The total drilling thrust (F1) is calculated by summing the forces at all the cutting edges of the drill [39]. Zhang et al. [39] interpreted a typical thrust force profile in micro-drilling, as shown in Figure 1a. In their study, they explained that in position 3, the major cutting edges have completely entered the hole, and the entire micro-drill is exerting thrust. In positions 1 and 2, the drill edges are gradually entering the hole and beginning to cut. Meanwhile, in positions 4 and 5, the tool is approaching the hole exit, which corresponds to the stage of burr formation [39].

Minimizing or eliminating burrs at the hole exit of the drill is crucial for achieving high-quality hole mounting. Burr formation depends on various factors, including the properties of the workpiece material, micro-drill geometry and material, and cutting conditions [42]. Hashimur et al. [42] reported that burr formation increases with the ductility of the materials, and the geometry of the micro-drill also affects the sizes of burrs. Both the thickness and height of burrs decrease as the point and helix angles increase. Furthermore, increases in feed rate and cutting speed result in larger burrs [42]. While burr shapes can vary, there are three basic types: uniform, transient, and crown burrs (Figure 1b) [43,44]. Uniform burrs have a relatively small and consistent height and thickness around the periphery of the hole, and their formulation should ideally be minimal. These burrs form when the first fracture occurs at the center of the hole. In the case of transient burrs, the fracture occurs simultaneously at the center of the hole and around the exit. Crown burrs are larger in size and have an irregular shape around the exit hole [44,45].

### Taguchi Methodology

Researchers often avoid the traditional full factorial design of experiments for optimization due to difficulties in determining the input-output relationship, as well as time constraints and financial losses in industrial and scientific investigations. The more experiments conducted, the higher the costs in terms of time and finances, leading to more complexity. Taguchi developed a method of designing experiments on how different parameters affect process performance with the goal of optimizing these parameters and enhancing product properties. Taguchi’s experimental design proposes using orthogonal arrays to identify the main process variables and their levels of response parameters [46]. These input parameters are variables within the process that affect the performance of responses. The design of orthogonal arrays for input parameters determines the number of conditions for each experiment. The selection of orthogonal arrays is determined by the number of parameters and the levels of variation for each parameter. The goal of the Taguchi method is to diminish manufacturing costs and inconsistent variability in manufacturing processes by defining the difference between the target value of the performance characteristic of a process (τ) and the measured value (y), which is a loss function, as shown below in Equation (1) [46].

(1) $$L\left(y\right)=\mathrm{kc}{\left(y-\tau \right)}^{2}$$

If the goal is for the performance characteristic value to be minimized, τ = 0, and the loss function is Equation (2):

(2) $$L\left(y\right)=\mathrm{kc}{\left(y\right)}^{2}$$

If the aim is for the performance characteristic value to be maximized, the loss function is Equation (3):

(3) $$L\left(y\right)=\frac{\mathrm{kc}}{{\left(y\right)}^{2}}$$

Engineering programs for Taguchi-designed experiments allow proper orthogonal arrays according to input parameters and their levels. The array selector assumes the same number of levels for each parameter, but this is not always the case. For instance, consider a situation where there are four parameters (P4) and three levels (S3) for each; a suitable orthogonal array is L9, which can be selected using the program’s array selector. Alternatively, consider a case with five parameters (A, B, C, D, and E), with four levels for A and B and two levels for C, D, and E. In this instance, a suitable orthogonal array is L16, selected by specifying the number of input parameters (P5) and the highest number of levels (4) [47]. Taguchi’s methodology identifies primary response variables via a selection of objective functions, often referred to as S/N ratios. The three objective functions most commonly used are “smaller is better,” “larger is better,” and “nominal is better.” These functions play a crucial role in the selection of S/N ratios in the Taguchi technique [48]. To determine the effect each parameter has on the output, the signal-to-noise ratio (S/N number) needs to be calculated for each experiment conducted. In equations 4, 5, and 6 below [49,50], y_i_ is the mean value, s_i_ is the variance, y_i_ is the value of the performance characteristic, i is the experimental number, N_i_ is the number of trials for an experiment i, and u is the trial number for a given experiment.

(4) $$S/N_{i}=10\log\frac{y_{i}^{2}}{s_{i}^{2}}$$

The following definition of the S/N ratio in Equation (5) should be calculated when minimizing the performance characteristic:

(5) $$S/N_{i}=-10\log\left(\overset{N_{i}}{\underset{u=1}{\sum }}\frac{y_{u}^{2}}{N_{i}}\right)$$

The following definition of the S/N ratio in Equation (6) should be calculated in the case of maximizing the performance characteristic:

(6) $$S/N_{i}=-10\log\left(\frac{1}{N_{i}}\overset{N_{i}}{\underset{u=1}{\sum }}\frac{1}{y_{u}^{2}}\right)$$

ANOVA is the analysis of variances used to compare continuous measurements and determine the significance of factors on measurements of output. *F* factors on measurements by looking at the relationship between a quantitative “response variable” and a proposed explanatory “factor.” A variable is correlated with one or more explanatory factors, typically using the F-statistic. From this F-statistic, the *p*-value can be calculated to see if the difference is significant. For example, if the *p*-value is low (*p*-value < 0.05 or 0.03), there is a low probability [43].

This research introduces an innovative perspective on the micro-drilling process of laminated Al–PE composite. The experimental investigation uncovers significant new findings regarding the impact of various drilling parameters, including point angle, tool diameter, coating, cutting speed, and feed, on the thrust force exerted during micro-drilling. The study not only highlights unique thrust force deviations during drilling of composite layers but also delves into the complexities of micromachining composites composed of materials with disparate properties. For instance, it elucidates a noticeable decrease in thrust force as the drill pierces through the softer PE layer. A distinguishing feature of this research lies in its robust quantitative analysis of the role of tool geometrical parameters in the micro-drilling process and their consequential effect on tool life, thereby highlighting the interaction between elements such as tool point angle and diameter. Unlike other studies on composites, this research provides crucial experimental and optimization results regarding thrust force and maximum burr height in the micro-drilling of special composite panels, where the upper and lower laminates are made of aluminum specially combined with a PE core through a rolling process. The primary objective here is to determine the thrust force characteristic in the PE layer of the Al–PE composite, followed by the lower Al layer after the top Al layer has been drilled, in addition to the effect of burr formation at the drilled hole exit. All responses were then used in an analysis of variance (ANOVA) to optimize the thrust force and burr height. Consequently, intriguing and distinct trust force curve characteristics and burr formations in micro-drilling were obtained using five variable parameters, differing from other studies.

## 3. Experimental Data and Methodology

**Figure 2:**
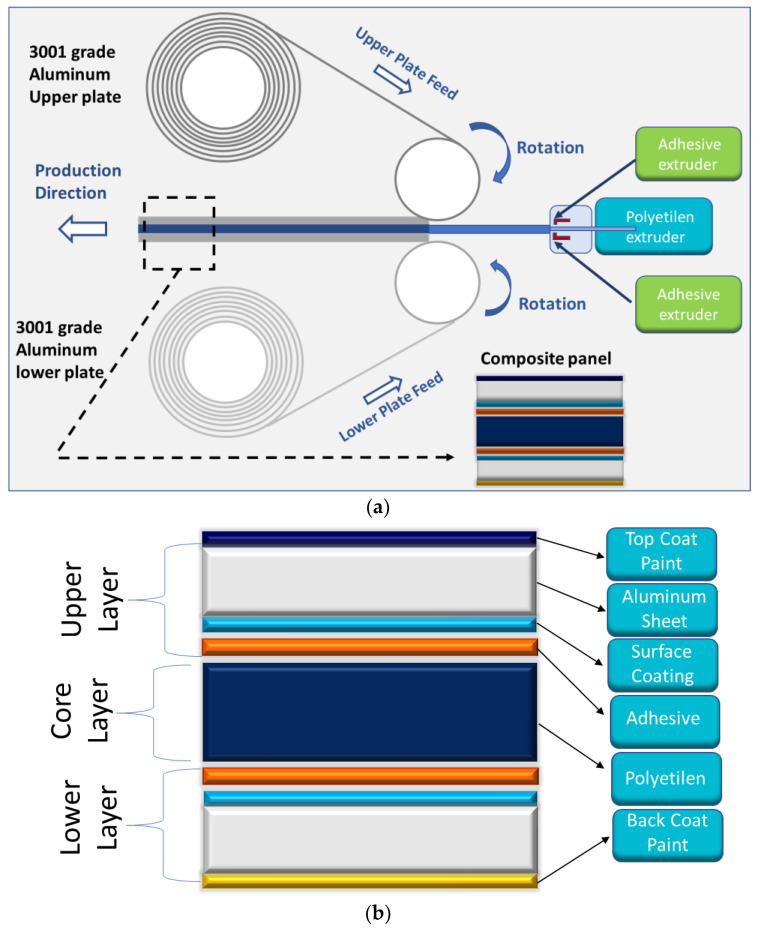
(**a**) Composite production line similar to rolling. (**b**) Testing the Al–PE composite material stuck layer.

The material used in the micro-drilling test is an aluminum composite panel that consists of upper and lower EN AW-3105 grade aluminum plates with a PE middle layer within the Al–PE composite stack. The Al–PE stack was manufactured by ASAŞ (Istanbul, Turkey) on a composite production line, a process similar to rolling, as shown in Figure 2. The laminated Al–PE composite was used as the material for the micro-drilling testing. The total thickness of the Al–PE composite panel is 4 mm, while the PE layers in the core of the composite have a thickness of 3 mm, an average elasticity modulus of 54 GPa, an average tensile strength of 212 MPa, and an average yield strength of 202 MPa. Table 1 displays the chemical composition of EN AW-3105-grade aluminum used for the upper and lower plates of the composite, with each AL layer having a thickness of 0.5 mm. Additionally, Table 2 presents the properties of the cutting tool used in the micro-drilling tests.

Micro-drilling parameters and their levels were designed using L16 Taguchi Orthogonal Array Design with Minitab, which can be seen in Table 2. A cutting speed with four levels of 10 m/min, 20 m/min, 30 m/min, and 40 m/min; a feed rate with four levels of 0.5 µm/rev, 1 µm/rev, 2 µm/rev, and 4 µm/rev; a tool diameter with two levels of 0.7 mm, 1 mm; a tool point angle with levels of 100° and 140°; and tool coating with two levels of TiAlN-coated and uncoated were used as input variables.

**Figure 3:**
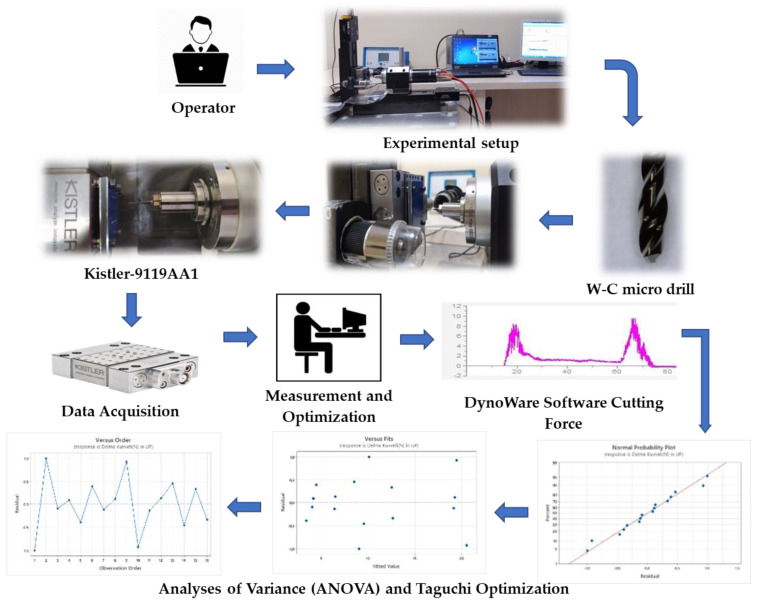
Details of the main experimental setup and process flow.

After conducting literature surveys, two levels were determined to be sufficient for understanding the overall trend of the effect of tool diameter, drill point angle, and coating on trust force and burr formation. However, because feed rate and cutting speeds are the main micro-drilling parameters that directly affect machining time and cost, as well as cutting force and thrust force, four-levels were preferred for these parameters. Experiments were conducted using L16 Taguchi Orthogonal Array Design, as shown in Table 3. The aim was to optimize the response of thrust force and burr formation involved in micro-drilling of Al–PE. Figure 3 shows the setup for the micro-drilling experiment. After designing the experiments, micro-drilling tests were performed under sixteen different conditions, and dry machining was carried out using a WC drill. Each experimental condition was repeated five times. The micro-machining center has a maximum power of 2.2 kW and a top spindle speed of 60,000 rpm. A Kistler 9119AA1 model mini dynamometer was used to measure the micro-cutting forces. The dynamometer provided high precision and helped to maintain a nearly constant ambient temperature. It is capable of measuring cutting forces up to ±40 N and, when calibrated for high loads, can also measure loads up to 4000 N.

Additionally, the characterization of hole burrs involves measuring and analyzing the shape of the burrs as well as determining their maximum size using high-resolution scanning electron microscopy (SEM). Burr height was measured by capturing an SEM image from the side, perpendicular to the hole. All the collected data were utilized to optimize the thrust force and minimize burr formation using ANOVA analysis with “smaller is better” functions. Important insights gained from ANOVA analysis included main effects plots for thrust force and burr formation, predicted curves, predictive modeling, surface graphics, residual plots, and SN ratios. These results were interpreted in the context of existing literature.

## 4. Results and Discussion

### 4.1. Thrust Force

**Figure 4:**
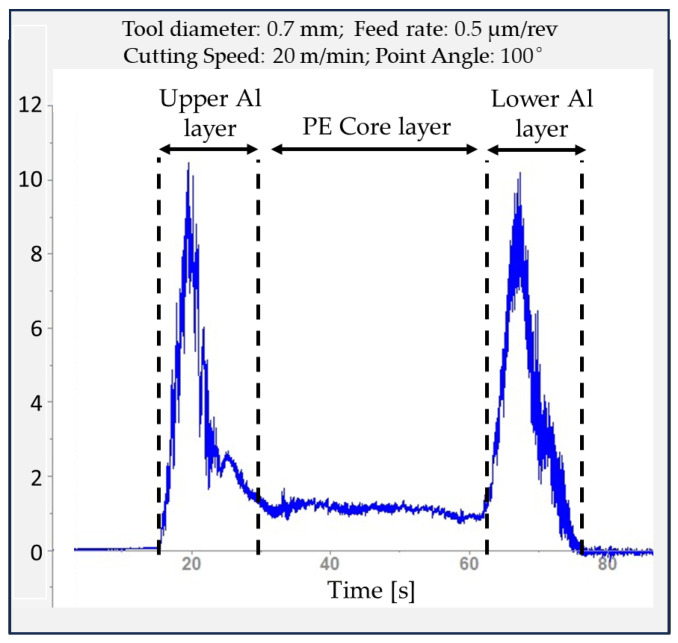
Thrust force curve samples obtained from the second experiment.

The micro-tool begins by cutting the upper Al layer of the laminated Al–PE composite, then enters the PE layer, and finally drills through the lower Al layer. This test produced interesting variations in thrust force that diverge from those observed in traditional materials. Figure 4 displays the thrust force variation over time for experiment number 2. As illustrated in Figure 4, when the cutting tool initially starts the cutting process, the force reaches its peak at 9.5 N. As the drill progresses into the PE core material, the thrust force drops sharply to 1.5 N. As the tool begins to drill through the lower Al layer, the thrust force increases once more, finally dropping to 0 N at the point where the drill exits.

The second drilling test was carried out with a 0.7 mm drill diameter, a 100° point angle, an uncoated tool, a 0.5 µm/rev feed rate, and a 20 m/min cutting speed. An increase in tool point and a decrease in tool diameter provide a decrease in thrust force. In particular, the tool life of small drills is strongly dependent on their geometry and is higher than a 100° point angle [51].

**Figure 5:**
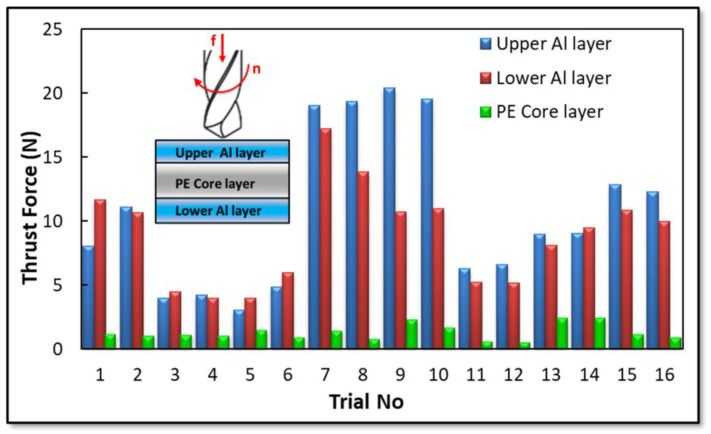
Maximum thrust forces for all micro-drilling conditions.

**Figure 6:**
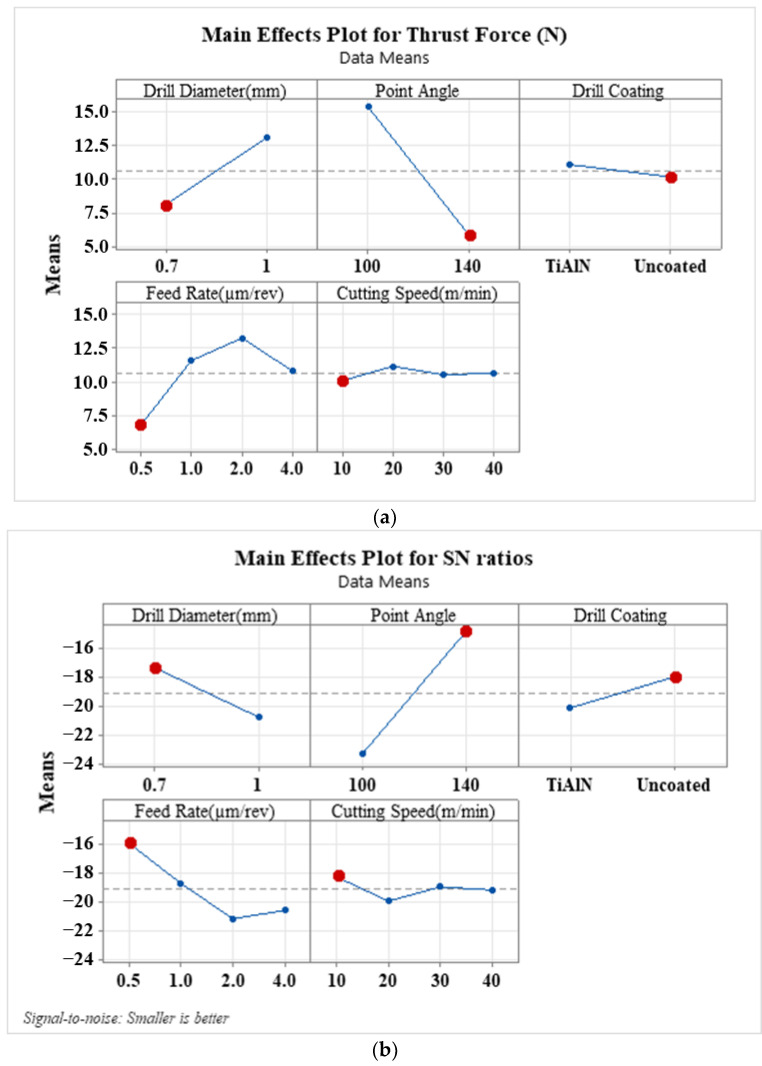
(**a**) Main effects plot and (**b**) SN ratio plot of the drilling parameters on the thrust force in the drilling of the upper layer of the composite when the drill enters.

**Figure 7:**
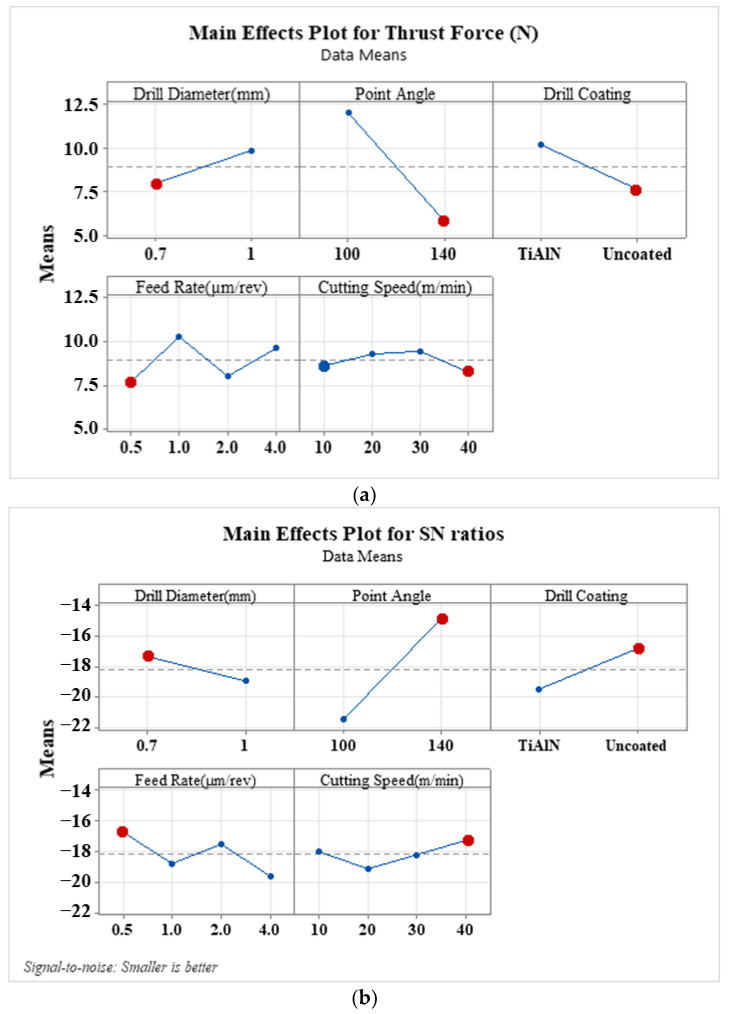
(**a**) Main effects plot and (**b**) SN ratio plot of drilling parameters on the thrust force in the drilling of the lower layer of the composite when the drill is close to the hole exit.

**Figure 8:**
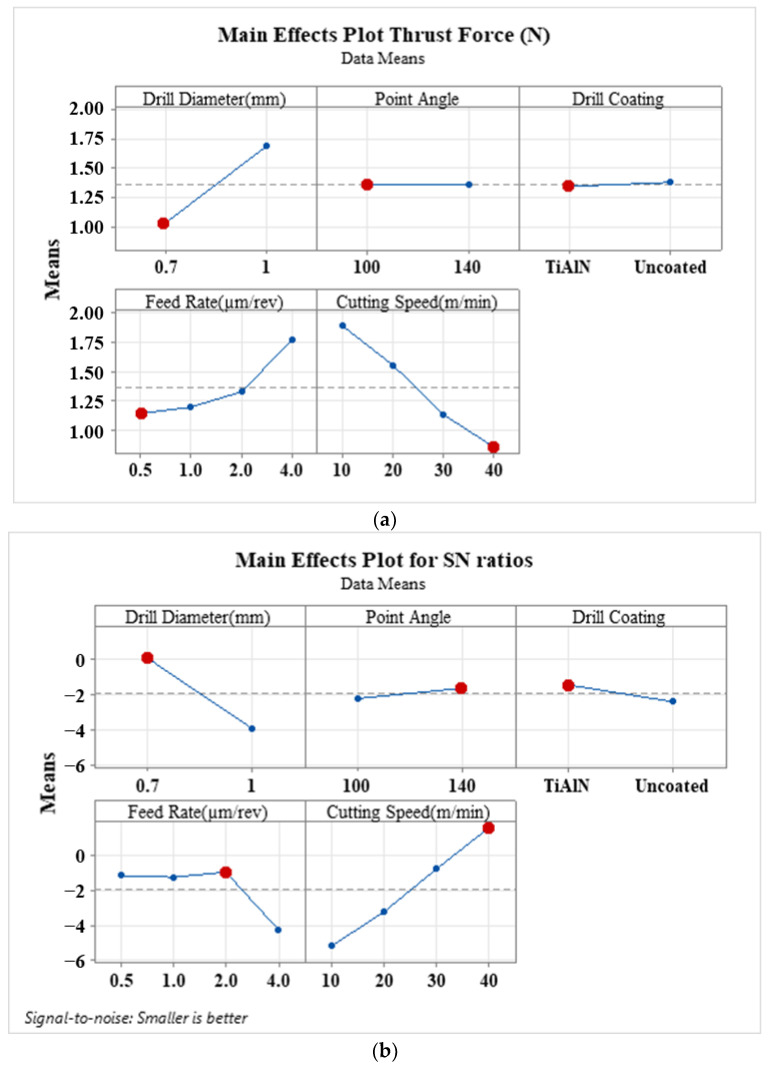
(**a**) Main effects plot and (**b**) SN ratio plot of drilling parameters on the thrust force in the drilling of the PE core layer of the composite.

In Figure 5, the maximum thrust force values for the upper and lower layers and the PE core layer of the laminated composite are given. As can be observed in Figure 5, the maximum thrust forces in trials 7, 8, 9, and 10 are higher than those of other trials. A possible reason for the high thrust force in trials 7, 8, 9, and 10 is the small tip angle (100°). A similar situation is observed in experiments 1, 2, 15, and 16. Generally, a decrease in point angle correlates with an increase in thrust force. Therefore, to identify the influence of various drilling parameters such as point angle, tool diameter, coating, cutting speed, and feed on the micro-milling of the Al–PE laminated composite, the main effects plot for thrust force was generated through variance analyses. The results are displayed in Figure 6, Figure 7 and Figure 8.

Figure 6 presents the main effects plot and SN ratios of drilling parameters on thrust force on the upper layer of the composite where the drill enters. According to Figure 6a, the tool point angle has the most important effect on the thrust force occurring in the upper layer of the composite during drill penetration. When the point angle increases from 100° to 140^o^, the thrust forces decrease. This is because a decrease in the point angle leads to an increase in chip thickness, which, in turn, increases the thrust force [52]. The tool diameter also significantly influences the thrust force; when the tool diameter changes from 0.7 mm to 1 mm, the thrust forces increase. Another study corroborated these findings, demonstrating that thrust force increases with an increased tool diameter [15]. The feed rate has a significant impact on thrust forces between 0.5 µm/rev and 2 µm/rev, but thrust forces slightly decrease with a feed rate of 4 µm/rev. Another study found that feed rate most significantly affects the main cutting force and surface roughness [53]. The use of an uncoated drill slightly reduces thrust force. A similar effect was observed for cutting speed. It can be concluded that a narrow point or tip angle and a feed rate of 0.5–1 µm/rev are preferable for stable shearing and reducing thrust force. The SN ratio plots shown in Figure 6b suggest that the lowest thrust force can be achieved from micro-drilling with an uncoated 140° point angle, a feed rate of 0.5 µm/rev, a cutting speed of 10 m/min, and a 0.7 mm drill diameter.

Figure 7 presents the main effects plot and SN ratio plots of drilling parameters on thrust force at the lower layer of the composite, where the drill exits. Additionally, the main effects plot of drilling parameters on thrust force during drilling of the PE core layer of the composite is displayed in Figure 8. According to Figure 7a, the tip angle exhibits a similar trend as seen during drilling of the upper layer. The most significant impact on the trust force during drilling of the lower layer among all parameters is observed when the tip angle increases from 100° to 140°. Consequently, a decrease in the tool point angle increases thrust forces. The effect of using an uncoated drill on thrust force at the hole exit is more pronounced than that of the upper layer. Therefore, the decreasing tendency of thrust force in micro-drilling with an uncoated W-C drill becomes more noticeable. It can be concluded that an uncoated drill is recommended for micro-drilling this type of composite structure. An upward trend in thrust force is observed with an increase in tool diameter. Slight downward trends in thrust force are observed with increasing feed rate and cutting speed. Rahamathullah et al. [30] demonstrated that the thrust force decreases with an increase in cutting speed during micro-drilling of polymer-based composites. According to the S/N ratio plots, the best cutting in the case of the drill close to the hole exit was provided with a 0.5 µm/rev feed rate, a 10 m/min cutting speed, 140° tip angles, and a 0.7 mm uncoated tool diameter. In Figure 8, the cutting speed has the greatest influence on thrust force during micro-drilling of the PE core layer, in contrast to the aluminum layers of the laminated composite. As the cutting speed increased from 10 m/min to 40 m/min, the thrust force sharply decreased. Conversely, thrust forces rise with an increase in feed rate, especially from 0.5 µm/rev to 4 µm/rev, as depicted in Figure 8a. Park et al. [54] suggested that a high cutting speed and a low feed rate are recommended for the production of a delamination-free, better surface finish while minimizing thrust force in drilling GLARE laminate. Moreover, tip angle and coating have almost no effect, and larger diameter tools exhibit a similar effect as observed in the drilling of the aluminum layers of the composite. The S/N ratios suggest the following optimal drilling parameters: a feed rate of 2 µm/rev, a cutting speed of 40 m/min, 140° tip angles, and 0.7 mm of the coated tool diameter. The variance analyses for thrust force occurring during the micro-drilling of each layer are, respectively, presented in Table 4, Table 5 and Table 6.

**Figure 9:**
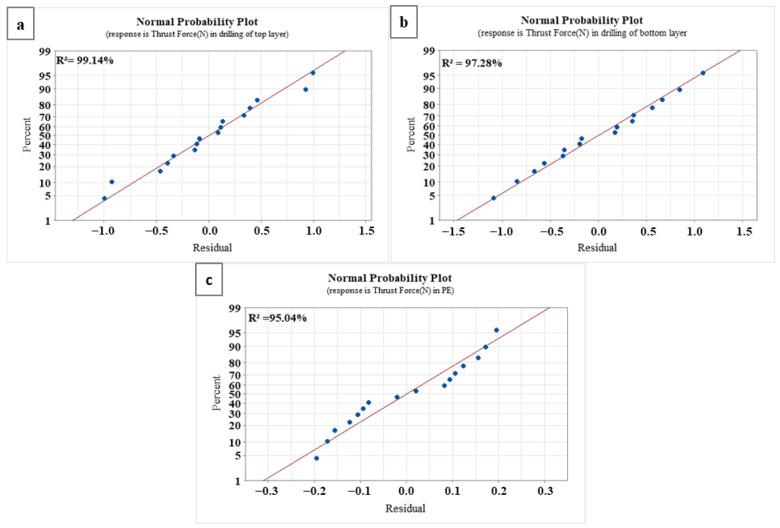
Probability plots for thrust force in the micro-drilling of each layer; (**a**) upper layer, (**b**) lower layer, (**c**) core layer.

According to Table 4, the point angle has the most significant effect at 64.54%, while the feed rate follows with a 15.93% contribution when the drill penetrates the composite. In addition, the contributions of cutting speed and coating to thrust force are 0.4% and 0.6%, respectively. Conversely, as the drill progresses into the PE core layer, the contributions of cutting speed and coating to thrust force are 45.32% and 17.71%, respectively, representing the most substantial and second-most significant effects, as shown in Table 5. The point angle has no contribution, and the coating contributes 0.09% to the thrust force. As indicated in Table 6, when the drill approaches the hole exit, the point angle accounts for 68.95%, the coating for 11.85%, the feed rate for 8.23%, and the cutting speed for 1.69% of the thrust force. The increase in drill diameter has an important effect on thrust force during drilling of the PE core layer. The probability plots for the thrust force of each layer are presented in Figure 9. Figure 9a shows that the normal probability percent for drilling the upper layer is 99.14%, whereas it is 97.8% for the lower layer, as shown in Figure 9b. Additionally, it is 95.04% for the PE core layer, as depicted in Figure 9c. These results confirm the critical importance of individual factors, such as point angle, feed rate, and cutting speed, at different stages of the drilling process. The dynamic interplay of drilling parameters as the drill transitions through layers of diverse material properties illustrates the complexity of such operations. It is also noteworthy that the impact of drill diameter increases significantly while drilling through the polymer layer, suggesting the need for careful selection and adjustment of this parameter to optimize force control [55].

### 4.2. Changing the Micro-Drilled Hole and Tool Diameter

**Figure 10:**
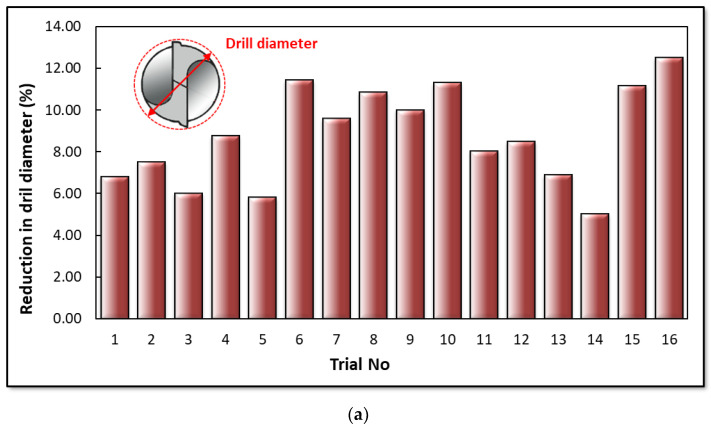

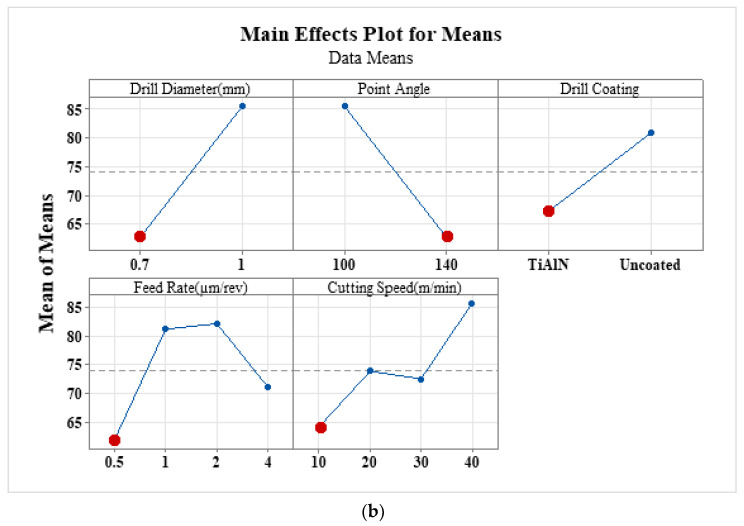
(**a**) Micro-tool diameter change (%) in 16 trials. (**b**) Main effects of drilling variables on the change in tool diameter.

In the micro-hole drilling process, hole quality depends on various factors, including the type of work material, cutting speed, feed rate, and tool geometry. During the process, changes in the hole diameter are caused by alterations in the tool diameter due to the abrasive wear mechanism. Consequently, in this study, changes in tool diameter were measured using scanning electron microscopy (SEM) after conducting experiments under identical cutting conditions. The values used in ANOVA (analysis of variance) are the averages of five measurements of both tool and hole diameters. Figure 10a illustrates the reduction in drill diameter. The results reveal that the least diameter change occurred in the 3rd, 5th, and 14th trials, while the most significant wear was observed in the 6th, 10th, and 16th trials. Maximum tool wear tends to occur on uncoated cutting tools with a smaller point angle. Figure 10b confirms this observation, showing that an uncoated drill and low point angle lead to an increase in tool wear. As the feed rate increased from 0.5 µm/rev to 1 µm/rev and the cutting speed reached 40 m/min, tool wear increased dramatically. For example, higher wear in the 16th trial occurred with a cutting speed of 40 m/min and an uncoated tool, whereas lower wear was seen in the third trial with a cutting speed of 30 m/min, a feed rate of 0.5 µm/rev, and a coated tool. Furthermore, ANOVA was performed to understand the effect of drilling parameters on tool diameter wear, as displayed in Table 7. According to this table, the tool point angle has the most significant effect (25.79%) on tool diameter wear. Following this, the feed rate emerges as a critical factor, having a 13.9% influence on tool wear. Tool wear increases with the enlargement of the drill diameter. Also, the positive effect of drill coating on drill wear was noted.

**Figure 11:**
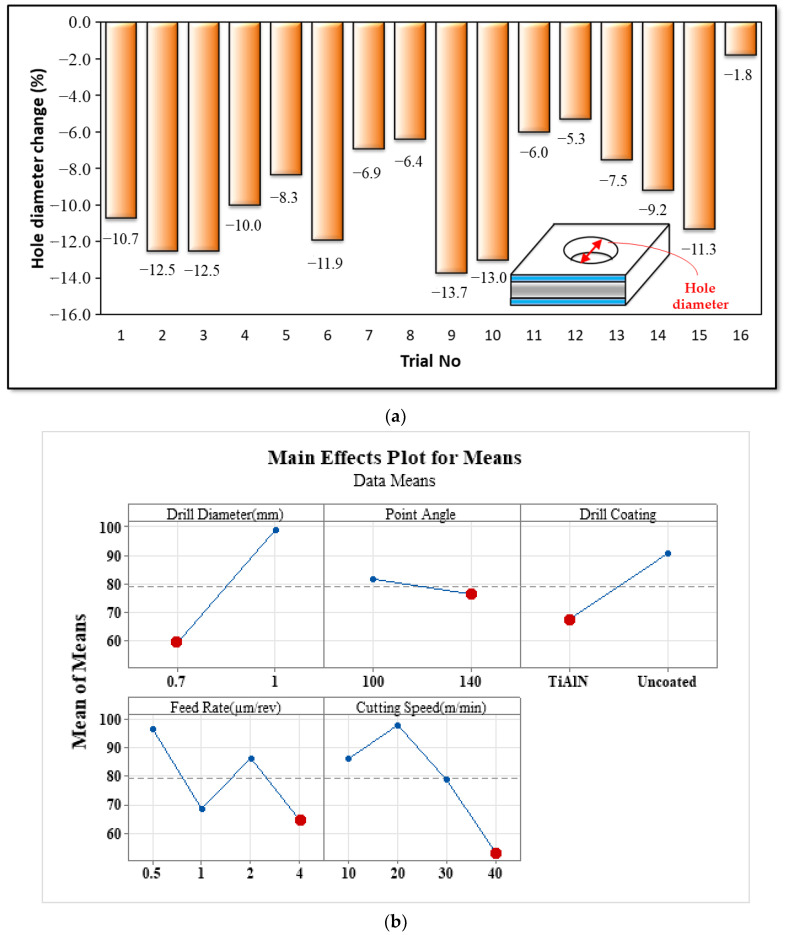
(**a**) Drilled hole diameter change for all micro-drilling tests. (**b**) Main effect of drilling parameters on the change in hole diameter.

A study [56] investigated the effect of cutting parameters on the hole diameter deviation. Unlike that study, the present investigation did not involve fiber composite, and therefore, the peel-up at the hole entrance did not occur. Consequently, hole diameter deviation was assessed by SEM measurements. In this study, Dh represents the hole diameters, and D corresponds to the drill diameters. The change in hole diameter was calculated using a basic deviation rate (Dh/Dort), and variance analyses were performed based on these measurements (Figure 11). As illustrated in Table 8, among drilling parameters, cutting speed had the most significant (25.15%) effect on hole diameter change. Furthermore, an increase in cutting speed and feed rate led to a decrease in hole diameter change. As drilling in a shorter time is allowed by increasing the cutting speed and feed rate, the change in hole diameter is minimized. As can also be seen from Table 8, the impact of the point angle on hole diameter deviation is minimal. Utilizing a coated tool resulted in a decrease in hole diameter variation, and an increase in drill diameter led to a rise in the amount of hole diameter deviation. A study [57] observed an increase in hole diameter deviation with an increase in tool diameter. In the same study, minimal hole diameter change was reported with the increase in feed rate and cutting speed. From this perspective, it can be said that the findings obtained from drilling Al–PE material under micro-conditions are consistent with the existing literature.

### 4.3. Effect of Drilling Parameters on Burr Formation

The exit burr height in drilling is much larger than the entrance burr height [57,58]. Therefore, in this study, the exit burr height was determined using SEM images. After each cutting condition, SEM images were obtained, and the burr height was measured. The average values of these measurements were used in the ANOVA analysis. The ANOVA variances are presented in Table 9.

**Figure 12:**
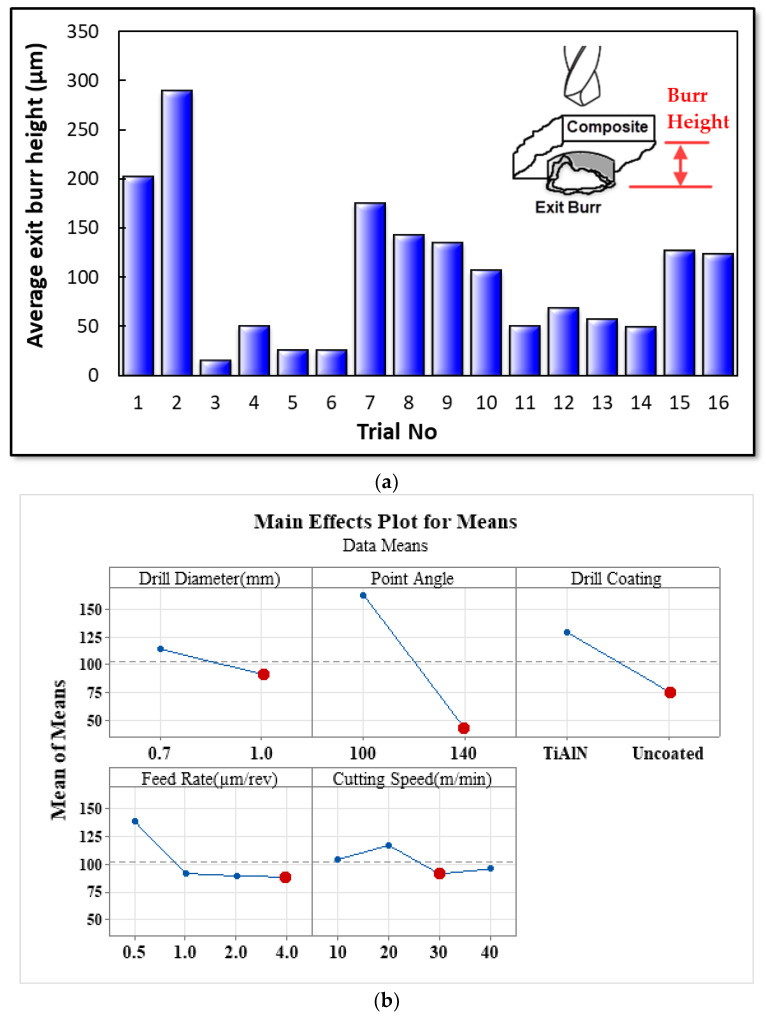
(**a**) Burr height at the hole exit for all tests. (**b**) Main effects of drilling variables on the burr height.

According to Table 9, the point angle has the highest influence on burr height at the hole exit. The AlTiN coating appears to negatively impact burr formation, while an increase in feed rate reduces burr height. The main effect of micro-drilling with different tool diameters (0.7 mm and 1 mm) is determined to be 2.41%. Figure 12a presents the maximum bur height during the first and second drilling trials. The drilling conditions were as follows: a micro-tool diameter of 1 mm, a point angle of 100°, a feed rate of 0.5 µm/rev, and a cutting speed of 10 m/min, with both AlTiN-coated and uncoated tools. Therefore, to minimize burr formation at the hole exit, an increase in cutting speed, feed rate, and point angle using an uncoated drill bit might be advantageous. Sorrentino et al. [58] reported that high-speed cutting and low-feed machining allow a significant reduction of the F_z_ component of the cutting force, which is responsible for any delamination/detachment issues in the material. Additionally, a decrease in spindle speed and high feed rate in the drilling of GLARE composite causes tool tip temperature, which causes problems of higher cutting force and critical resin temperature in polymeric composite materials [59,60].

As stated earlier, according to Table 9, the point angle has a major impact on burr height at the hole exit. A reduction in the point angle results in a decrease in chip thickness, which complicates chip breakage and increases burr formation. Chen et al. [61] reported that a negative point angle in the cutting process promotes the formation of a negative shear deformation zone at the exit surface, thereby increasing the exit burr. Therefore, increasing the point angle can reduce burr formation. It is also observed that the AlTiN coating has a detrimental effect. As the coating material acts as a thermal barrier, more heat from the cutting zone transfers to the workpiece. This leads to the thermal softening of the workpiece, thereby increasing the formation of burrs. Similar effects were observed in [62], where burr height and width were increased due to the thermal softening of the workpiece. An increase in feed rate also contributed to the reduction in burr height. The increased feed rate leads to greater chip thickness, thereby facilitating chip breakage and resulting in smaller burrs. Another finding is that the effects of cutting speed and tool diameter on burr formation are considerably less significant. Based on the findings presented in Figure 12b, it is recommended to use uncoated drill bits and high cutting speeds, feed rates, and point angles to minimize burr formation at the hole exit.

**Figure 13:**
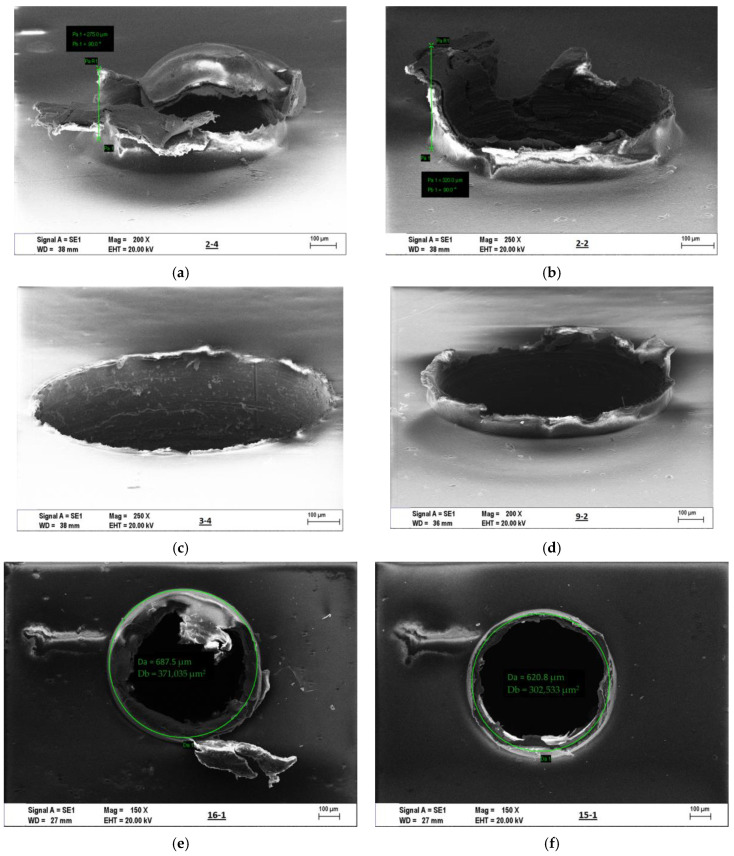

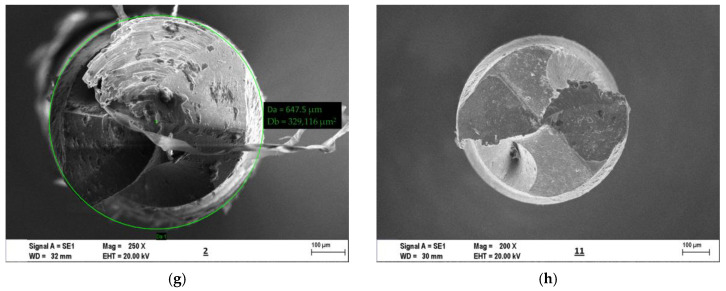
SEM screens selected from among all results: sample of the exit burr (**a**–**d**), hole diameter changes (**e**,**f**), and chip adhesion on the drilling tool (**g**,**h**).

**Figure 14:**
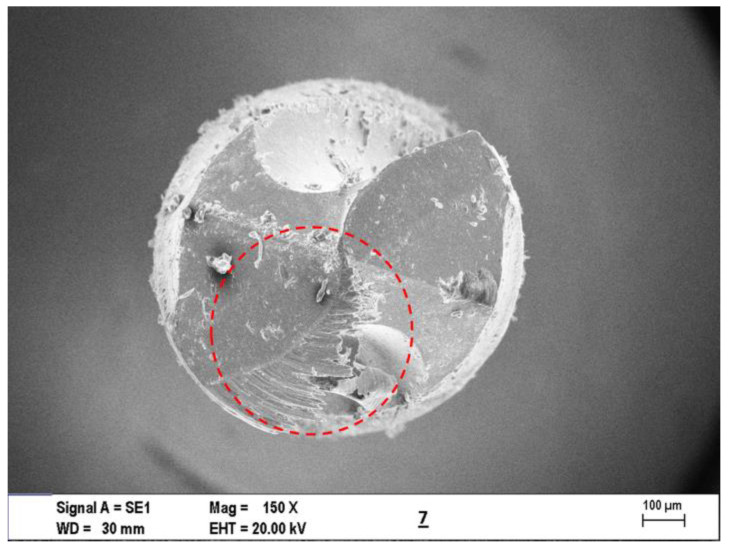
SEM photography for extreme chip adhesion on the major cutting edge and nose area observed in the seventh condition.

In Figure 13, the SEM screens selected from among all results were given. Figure 13a,b presents the highest average burr height measured from five specimens in the second micro-drilling test. As can be seen, the hole exit is quite a bur and not clean. Therefore, there is a need for de-burring after micro-drilling laminate composite. Figure 13c shows the lowest average burr formation at the hole exit with the third micro-drilling condition. This sample hole exit is the cleanest and has almost no de-burring process. Figure 13d indicates that this is the hole where the highest thrust force occurred. Figure 13e shows a sample of the hole diameter measurement applied to all drilled holes. All average hole diameters were measured in five different holes. Another hole diameter is lower than the drill diameter, depending on the decrease in tool diameter with wear. This state was shown as an example in Figure 13f. In the second experimental condition, the burr occurred at its maximum, and chip adhesion was observed to the micro-drill. On the other hand, chip adhesion concentration has been seen in the core diameter and nose area of the micro-tool in drilling with the ninth condition, which is given in Figure 13g. This phoneme increases the thrust force by preventing the progress of the drill in the composite structure. Similarly, an extremely high chip adhesion (red dotted circle in Figure 14) on the major cutting edge and nose area was observed in the seventh condition, which is one of the experiments in which the thrust force maximum was given in Figure 14.

## 5. Conclusions

The purpose of this study was to understand the micro-drilling tendencies of Al–PE laminate composites. Micro-drilling tests were carried out using the Taguchi L16 orthogonal array and ANOVA analyses. These analyses included measurements of thrust force, exit burr height, drilling tool diameter, and drilled hole diameter. Based on the experimental results, the following findings were made:

➢ When drilling the Al–PE material, the maximum thrust force was obtained in the aluminum sheets, while the thrust force in the PE material was minimal. This does not change with cutting parameters and tool geometry.
➢ The point angle of the tool has the most significant effect (64.54%) on the thrust force. The effect of feed rate on the thrust force is 15.93%, while cutting speed and tool coating have an effect of 0.4% and 0.6%, respectively.
➢ The point angle has the most significant effect on the change in tool diameter (25.79%), while the feed rate has a 13.9% effect on tool wear. The coated tool, although reducing tool wear, was found to have the least effect on diameter change (9.29%).
➢ The cutting speed has the greatest effect on the change in hole diameter (25.15%). Increasing the cutting speed and feed rate also reduced the hole diameter variation.
➢ Among the cutting parameters, the effect of feed rate on hole diameter variation is 16.13%. However, while the effect of the AlTiN coating is 12.38%, it can be said that the tool point angle has a negligible effect (0.64%).
➢ The point angle has the greatest effect (67.80%) on the burr height at the hole exit, and the AlTiN coating caused the burr height to increase. This indicates that the heat in the cutting zone is transferred to the workpiece, and thermal softening of the workpiece occurs.
➢ In light of the results, it can be said that the optimum cutting condition in terms of minimum thrust, burr height, and minimum diameter change is the fifth experiment.

## Figures and Tables

**Table 1:** Chemical composition of upper and lower aluminum plates (EN AW-3105).

Si	Fe	Cu	Mn	Mg	Cr	Ni	Zn	Ti
0.6	0.7	0.3	0.3–0.8	0.2–0.8	0.2	-	0.4	0.1

**Table 2:** Taguchi orthogonal array design (L16 (4^2^ 2^3^)).

Symbols	Micro-Drilling Parameters	Levels			
		1	2	3	4
A	Drill Diameter (mm)	0.7	1	-	-
B	Point Angle (°)	100	140	-	-
C	Drill Coating	TiAlN Coated	Uncoated	-	-
D	Feed Rate (µm/rev)	0.5	1	2	4
E	Cutting Speed (m/min)	10	20	30	40

**Table 3:** Micro-drilling parameters and levels.

Trial No. L16	Micro-Drilling Parameters (Inputs)				
	A (mm)	B (°)	C	D (µm/rev)	E-Cutting Speed (m/min)
1	0.7	100	Coated	0.5	10
2	0.7	100	Coated	0.5	20
3	1	140	Uncoated	0.5	30
4	1	140	Uncoated	0.5	40
5	0.7	140	Uncoated	1	10
6	0.7	140	Uncoated	1	20
7	1	100	Coated	1	30
8	1	100	Coated	1	40
9	1	100	Uncoated	2	10
10	1	100	Uncoated	2	20
11	0.7	140	Coated	2	30
12	0.7	140	Coated	2	40
13	1	140	Coated	4	10
14	1	140	Coated	4	20
15	0.7	100	Uncoated	4	30
16	0.7	100	Uncoated	4	40

**Table 4:** Analysis of variance for thrust force in the drilling of the upper layer.

Source	DF	Seq SS	Contribution	Adj SS	Adj MS	F-Value	*p*-Value
Drill Diameter (mm)	1	97.719	17.68%	97.719	97.719	123.82	0.000031
Point Angle (°)	1	356.817	64.54%	356.817	356.817	452.12	0.000001
Drill Coating	1	3.326	0.60%	3.326	3.326	4.21	0.085890
Feed Rate (µm/rev)	3	88.063	15.93%	88.063	29.354	37.19	0.000285
Cutting Speed (m/min)	3	2.195	0.40%	2.195	0.732	0.93	0.483003
Error	6	4.735	0.86%	4.735	0.789		
Total	15	552.857	100.00%				

**Table 5:** Analysis of variance for thrust force in the drilling of the PE core layer.

Source	DF	Seq SS	Contribution	Adj SS	Adj MS	F-Value	*p*-Value
Drill Diameter (mm)	1	1.73224	31.93%	1.73224	1.73224	38.63	0.000801
Point Angle (°)	1	0.00021	0.00%	0.00021	0.00021	0.0047	0.947546
Drill Coating	1	0.00488	0.09%	0.00488	0.00488	0.11	0.752618
Feed Rate (µm/rev)	3	0.96071	17.71%	0.96071	0.32024	7.14	0.020933
Cutting Speed (m/min)	3	2.45882	45.32%	2.45882	0.81961	18.28	0.002019
Error	6	0.26902	4.96%	0.26902	0.04484		
Total	15	5.42589	100.00%				

**Table 6:** Analysis of variance for thrust force in the drilling of the lower layer.

Source	DF	Seq SS	Contribution	Adj SS	Adj MS	F-Value	*p*-Value
Drill Diameter (mm)	1	14.489	6.56%	14.489	14.489	14.48	0.008909
Point Angle (^o^)	1	152.386	68.95%	152.386	152.386	152.32	0.000017
Drill Coating	1	26.196	11.85%	26.196	26.196	26.18	0.002184
Feed Rate (µm/rev)	3	18.190	8.23%	18.190	6.063	6.06	0.030133
Cutting Speed (m/min)	3	3.743	1.69%	3.743	1.248	1.25	0.372784
Error	6	6.003	2.72%	6.003	1.000		
Total	15	221.006	100.00%				

**Table 7:** Analysis of variance for tool diameter wear in the drilling of the composite.

Source	DF	Seq SS	Contribution	Adj SS	Adj MS	F-Value	*p*-Value
Drill Diameter (mm)	1	2060.0	26.15%	2060.0	2060.0	12.09	0.013
Point Angle	1	2031.5	25.79%	2031.5	2031.5	11.92	0.014
Drill Coating	1	731.8	9.29%	731.8	731.8	4.30	0.084
Feed Rate (µm/rev)	3	1094.9	13.90%	1094.9	365.0	2.14	0.196
Cutting Speed (m/min)	3	936.4	11.89%	936.4	312.1	1.83	0.242
Error	6	1022.2	12.98%	1022.2	170.4		
Total	15	7876.9	100.00%				

**Table 8:** Analysis of variance for changes in hole diameter.

Source	DF	Seq SS	Contribution	Adj SS	Adj MS	F-Value	*p*-Value
Drill Diameter (mm)	1	6275.4	36.67%	6275.4	6275.4	24.36	0.003
Point Angle	1	109.6	0.64%	109.6	109.6	0.43	0.538
Drill Coating	1	2119.0	12.38%	2119.0	2119.0	8.23	0.028
Feed Rate (µm/rev)	3	2760.0	16.13%	2760.0	920.0	3.57	0.086
Cutting Speed (m/min)	3	4305.2	25.15%	4305.2	1435.1	5.57	0.036
Error	6	1545.6	9.03%	1545.6	257.6		
Total	15	17114.7	100.00%				

**Table 9:** Analysis of variance for burr height at the hole exit in the drilling of the composite.

Source	DF	Seq SS	Contribution	Adj SS	Adj MS	F-Value	*p*-Value
Drill Diameter (mm)	1	2047	2.41%	2047	2047.0	2.36	0.17538
Point Angle (°)	1	57,627	67.80%	57,627	57,627.0	66.44	0.00018
Drill Coating	1	11,352	13.36%	11,352	11,351.6	13.09	0.01113
Feed Rate (µm/rev)	3	7224	8.50%	7224	2408.1	2.78	0.13278
Cutting Speed (m/min)	3	1539	1.81%	1539	513.1	0.59	0.64290
Error	6	5204	6.12%	5204	867.3		
Total	15	84,993	100.00%				

## Data Availability

Not applicable.
